# Applying the Small-Area Estimation Method to Estimate a Population Eligible for Breast Cancer Detection Services

**Published:** 2007-12-15

**Authors:** Kirsten Knutson, Weihong Zhang, Farzaneh Tabnak

**Affiliations:** California Department of Public Health, CDIC/Cancer Detection Section; California Department of Public Health, Cancer Detection Section, Sacramento, California; California Department of Public Health, Cancer Detection Section, Sacramento, California

## Abstract

**Introduction:**

Populations eligible for public health programs are often narrowly defined and, therefore, difficult to describe quantitatively, particularly at the local level, because of lack of data. This information, however, is vital for program planning and evaluation. We demonstrate the application of a statistical method using multiple sources of data to generate county estimates of women eligible for free breast cancer screening and diagnostic services through California's Cancer Detection Programs: Every Woman Counts.

**Methods:**

We used the small-area estimation method to determine the proportion of eligible women by county and racial/ethnic group. To do so, we included individual and community data in a generalized, linear, mixed-effect model.

**Results:**

Our method yielded widely varied estimated proportions of service-eligible women at the county level. In all counties, the estimated proportion of eligible women was higher for Hispanics than for whites, blacks, Asian/Pacific Islanders, or American Indian/Alaska Natives. Across counties, the estimated proportions of eligible Hispanic women varied more than did those of women of other races.

**Conclusion:**

The small-area estimation method is a powerful tool for approximating narrowly defined eligible or target populations that are not represented fully in any one data source. The variability and reliability of the estimates are measurable and meaningful. Public health programs can use this method to estimate the size of local populations eligible for, or in need of, preventive health services and interventions.

## Introduction

At a time when more than 16% of the population of the United States (more than 47 million people) lack insurance coverage for basic medical services, an important function of public health is to provide the underserved and people disproportionately affected by disease with access to preventive health services (1). To reach these people effectively, public health programs are best implemented locally, in counties or cities (2). Estimating the population eligible or targeted for specific services is often difficult at the local level, however, because of lack of data (3-7).

Because public health programs or interventions are usually tailored to improve the health of specific underserved or high-risk groups, an individual may have to meet particular criteria (e.g., be a woman aged 40 years or older with no health insurance) to be eligible for program services or may have to belong to a target group characterized by the intervention (e.g., women with low personal income at risk for pregnancy). Decennial census and intercensal population projections provide summary counts of local populations by various demographic characteristics, but these sources rarely contain data corresponding to the narrowly defined criteria that usually describe eligibility for public health programs. Public health surveys collect a wide range of information, but they, too, may not contain the necessary data for generating reliable estimates of local populations (3,4,6-10). In fact, many surveys conducted statewide have so few respondents that even state estimates of small eligible or target populations are unreliable.

Because an epidemiologic description of the eligible or target population is essential to developing and operating a public health program or intervention (11), the problem of insufficient data must be addressed. Reliably defining the service-eligible population tells program planners how many people are eligible for services, who these people are, and where they live and is central to such activities as projecting costs, preparing budget proposals, and justifying funding requests. Equally important, reliable estimates help planners determine the portion of the eligible population that the program cannot serve, given available resources. If funding is insufficient to reach all eligible people, estimates of subgroups of the eligible population, as defined by various demographic, geographic, or high-risk characteristics, enable a program to identify priority target groups, establish realistic enrollment goals, and request appropriate funding (12). Reliable estimates also provide evidence for program growth and infrastructure development and provide essential data for decision making about program policy and resource allocation and for monitoring and evaluating a program's effectiveness (13,14).

We demonstrate how the small-area estimation method was used by the California Department of Public Health to estimate the size of local populations that are eligible for free breast cancer screening and diagnostic services through the state's Cancer Detection Programs: Every Woman Counts (CDP:EWC). Although this method can be used in many ways, including to adjust for census undercounts and to estimate populations in political districts, it is presented here as a reliable approach to resolving the problem encountered by public health programs of precisely estimating local populations eligible for preventive health services and interventions when no single data source is adequate for the task (4,8,15).

## Methods

### Data

We used two data sources in our analysis. The primary source was the California Women's Health Survey (CWHS), an annual population-based telephone survey that is coordinated and conducted by the California Department of Public Health and funded in collaboration with the California Department of Mental Health, the California Department of Alcohol and Drug Programs, Lumetra (formerly California Medical Review, Inc), the California Department of Social Services, and the Public Health Institute. The survey data are intended to provide state estimates on women's health behaviors and attitudes.

CWHS employs a screened random-digit–dialed sampling method to select households to be called. Women aged 18 years or older residing in a contacted household are eligible to participate in the survey. From 1997, when the survey began, through 2003, CWHS conducted an annual average of 4147 interviews statewide, with an annual average (upper-bound) response rate of 72.9%. Additional information on methods used by CWHS is available from the California Department of Public Health, Survey Research Group (16).

To obtain a sample size appropriate for stratifying by small geographic area, we aggregated CWHS data from 1998 through 2003. Our initial sample consisted of 14,284 women aged 40 years or older who were interviewed by CWHS during this 6-year period. We excluded from analysis 445 respondents (3.1%) who did not complete the interview; 636 (4.5%) who responded "don't know" to, or refused to answer, questions necessary to determine health insurance and poverty status; and 23 (0.2%) who responded "don't know" to, or refused to answer, questions used to determine racial/ethnic group, marital status, education level, or county of residence. Our final sample size was 13,180.

The second data source was Census 2000, Summary File 3 (SF 3) (17), which contains socioeconomic and housing information collected from a sample (about 1 in 6 households) of the approximately 19 million housing units nationwide that received the Census 2000 long-form questionnaire. For each of the 58 California counties, we extracted data from SF 3 that corresponded with the socioeconomic characteristics identified as possibly associated with eligibility for CDP:EWC (15).

### Measures

The dependent measure for this analysis was a binary variable representing eligibility for CDP:EWC services. Using CWHS data, we derived eligibility status for each respondent in our final sample of women aged 40 years or older, according to self-reported poverty and health insurance status. Respondents were considered eligible if they reported having an annual household income at or below 200% of the federal poverty level and having neither Medicaid nor Medicare. All other women were categorized as ineligible for services.

**Individual Measures**

From CWHS data, we extracted information on each woman's county of residence and derived each woman's racial/ethnic group, education level, and marital status.

CWHS questions about racial/ethnic group varied over the study period. To determine ethnicity, in some years CWHS asked women if they were Hispanic and, in other years, if they were Latina, so that the same ethnicity information was collected each year, even though the wording of the question changed over time in accordance with federal guidelines for the collection of these data (18). From 1998 through 2000, women chose a single race from seven racial groups that were read to them. From 2001 through 2003, women were asked to identify their race in the same way, but they could choose one or multiple racial groups. Women who reported being of multiple races were then asked to choose the group with which they most identified. We categorized as Hispanic (an ethnicity) all respondents who identified themselves as either Hispanic or Latina, regardless of their racial group. We categorized non-Hispanic respondents by their reported racial group, with respondents giving multiple races in the 2001 through 2003 surveys being categorized according to the racial group with which they most identified.

We divided education status into two categories: *high school or less *for respondents who reported no more education than completing high school or obtaining a GED (general education development) certificate, and *college or more *for those who reported any amount of college or technical school.

We also divided marital status into two categories: *married/partnered *for respondents who reported being married or separated and *unmarried/unpartnered *for those who reported being a member of an unmarried couple, divorced, widowed, or never married.

**County Measures**

We extracted county data on per capita income from Census 2000, SF 3, Table P82 and median household income from SF 3, Table P53. Both variables represented county residents of all ages.

We defined the county unemployment rate as the proportion of women in the labor force aged 35 to 64 years (available age group) who were unemployed at the time of census and derived this information from SF 3, Table P35. The denominator comprised all women in a county in this age group.

We derived the percentage of women living in poverty in each county from data in SF 3, Table PCT49, by dividing the number of women aged 35 to 64 years (available age group) who were living below the federal poverty level by the total number of women in a county in this age group.

All variables were continuous.

### Statistical Analysis

We used the small-area estimation method (4,8,19,20) to generate regression-based estimates of the proportion of women eligible for CDP:EWC services. To demonstrate the usefulness of this method for estimating local and sparse target populations, we calculated estimates of service-eligible women by county and by racial/ethnic group within each county. We performed the regression analysis using SAS Version 8 and a corresponding macro, GLIMMIX, (SAS Institute Inc, Cary, North Carolina) (21).

To obtain the parameter estimates, we fitted the model using the restricted/residual pseudolikelihood method (22). All county variables were standardized to observe the mean and standard deviations. We included individual and county variables as covariates in a generalized, linear, mixed-effect model with eligibility status as the outcome variable. To account for the variation not explained by the regression variables, a county random effect variable, a*_i_*, was included in the model:

Logit [p(yij=1ai)]=xijb+ai

In the model, *X_ij_* is the *j*th observation in county *i* for racial/ethnic group, educational level, marital status, unemployment rate, percentage of women living in poverty, median household income, and the interaction terms between these variables; *y_ij_* is a Bernoulli random-response variable with probability *p_ij_*; and a*_i_* is assumed to be normally distributed with a mean of zero and a variance equal to σ^2^.

During preliminary analysis, we compared the Akaike Information Criterion (AIC) values of the model variables to assess their relative contribution to the model. Education level and marital status, which had the lowest AIC values and did not contribute to the model selection, were not included as variables. The racial/ethnic and county variables and the interaction terms were maintained in the preliminary model.

Next we used backwards selection (23) to determine which variables and interactions of variables to select for the final model. To increase predictability, we set the selection criteria for the model at a = 0.30, rather than at a lower level (24). The variables representing unemployment rate, percentage of women living in poverty, median household income, each racial/ethnic group, and significant interaction terms remained in the final model (Table 1).

We used the Monte Carlo method (25) to estimate the proportion of eligible women in each racial/ethnic group in each county and the bootstrap method (26) to calculate the standard error of the estimated proportions in each racial/ethnic group and in all races combined. We calculated 95% confidence intervals for each standard error. We computed the coefficient of variation (CV) to assess the reliability of the estimated prevalence points (4,27) and considered proportions with a CV greater than 0.23 unreliable. All county estimates were found to be reliable.

## Results

The estimated county percentages of eligible women varied from a minimum of 5.5% (Marin County) to a maximum of 35.3% (Imperial County) (Table 2). The estimated percentage at the 25th percentile was 11.1%; at the 50th percentile (median), 13.6%; and at the 75th percentile, 15.9%. The mean of the estimates was 13.9%. The estimated proportions were not normally distributed, but skewed to the right.

The small-area estimation method yielded a wide range and considerable variability in the estimated proportions across counties (Figure). The estimated proportions of eligible Hispanic women varied more than did those of women of other races. Even so, the range of estimates across counties in each racial group was more than 10%. Imperial County, one of the outliers in the figure, had the highest proportion of eligible women of all races combined. In the second outlier, Del Norte County, an estimated 23.2% of black women aged 40 years or older were eligible for CDP:EWC services.

**Figure:**
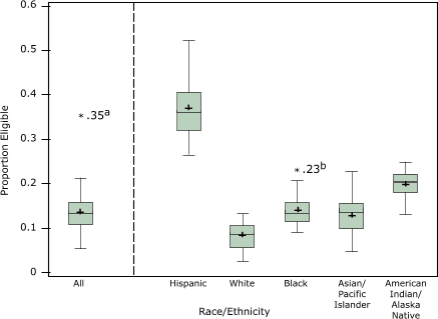
Estimated proportions of women aged 40 years or older in California counties eligible for breast cancer screening and diagnostic services through Cancer Detection Programs: Every Woman Counts, by racial/ethnic group, 1998–2003. Asterisk (*) indicates value suspected as outlier; plus (+), mean of county proportions. *Note.* The bottom line of each box represents the 25th percentile of the estimated proportions; the middle line, the 50th percentile (median), and the top line, the 75th percentile. The endpoints of the whiskers are the most extreme values not identified as suspected outliers. We identified as suspected outliers county proportions exceeding the 75th percentile plus 1.5 times the interquartile range, or falling short of the 25th percentile minus 1.5 times the interquartile range.

**Table:** 

NA indicates not applicable.

a Imperial County.

b Del Norte County.

Estimated proportions of eligible women showed considerable variability by race within counties (data not displayed graphically). In every county, the estimated proportion of women aged 40 years or older who were eligible for CDP:EWC services was higher for Hispanics than for whites, blacks, Asian/Pacific Islanders, and American Indians/Alaska Natives. In Imperial County, an estimated 45.2% of Hispanic women aged 40 years or older were eligible for program services, compared with 18.6% of black women, 15.8% of American Indian/Alaska Native women, 12.1% of white women, and 10.2% of Asian/Pacific Islander women. In Los Angeles County, which is the most populous of the state's counties and had the fifth highest estimated proportion of eligible women (20.0%), an estimated 40.2% of Hispanic women, 19.2% of American Indian/Alaska Native women, 15.7% of black women, 13.3% of Asian/Pacific Islander women, and 8.4% of white women were eligible. These proportions were all reliable. Some of the estimated proportions of eligible Asian/Pacific Islander and American Indian/Alaska Native women were not reliable, however, because of the small sample sizes in the CWHS for women in these racial/ethnic groups.

## Discussion

When calculating reliable estimates directly from survey or population data is not possible, the ability to combine multiple sources of data, each with different facets of the necessary information, is a strength of the small-area estimation method. In our example, available survey data contained information corresponding to CDP:EWC eligibility criteria, but they were appropriate only for state estimates. With the small-area estimation method, we were able to supplement statewide survey data with community census data by means of statistical modeling and produce reliable estimates for each California county.

Although the term *small-area estimation* suggests that this method is used to estimate populations living in small geographic areas, this method is also useful in identifying sparse target populations. Describing the distribution of a narrowly defined characteristic in racial/ethnic groups is a common problem because of the small number of people in some of these groups. With small-area estimation, however, we were able to calculate reliable estimates of service-eligible women in five racial/ethnic groups for most California counties.

Public health professionals have synthetically calculated local estimates when data with an adequate sample size to directly calculate local estimates are unavailable (3,4,9,10). In our demonstration, for example, we could have calculated a direct estimate of the proportion of eligible women in California from CWHS data and then multiplied each county's census population by this proportion to estimate the local numbers of eligible women. The resulting estimates would be based on the assumption that the demographic characteristics that define program eligibility are present in every county in the same proportion as they are in the state (4,8). This would be a poor assumption, however, because the synthetic method would estimate that 16.3% of women aged 40 years or older in each county were eligible for CDP:EWC services, whereas the small-area estimation method that we used yielded widely varying estimated proportions by county.

Another benefit of the small-area estimation method is that variability and reliability can be measured, and these statistics are informative. Although the standard error and confidence interval can be calculated for each synthetically generated point estimate (i.e., proportion of eligible women), these measures are not meaningful because the estimates themselves are limited by the flawed assumption we have described.

As with any means of estimation, however, obtaining statistically reliable results depends on factors such as sample size. When generating local estimates in the absence of sufficient local data, the small-area estimation method allows the researcher to borrow strength from available data (9,20). For some sparse local populations, however, no amount of supplemental information can compensate for the small number of survey respondents sampled, and model-based estimates for these populations will be unreliable.

A major limitation of small-area estimation statistics is that diagnostics for checking nonlinear models are few and not well-developed (8). Even so, comparing model-based with directly calculated survey-based estimates of the target population in the large area (i.e., the aggregate of local areas) can provide some indication of the performance of a model (28). For example, our method estimated that 15.3% of California women aged 40 years or older were service-eligible, whereas the direct method yielded an estimate of 16.3%. For practical purposes, the two estimates are similar, and without a gold standard, observing similar values resulting from two different methods can be a qualitative confirmation of methods and analysis. Although the statistical method that we used has been validated (4,8), a model-based overall estimate that was vastly different from the survey-based direct estimate would be a signal to the researcher to reassess the analysis.

California's CDP:EWC program has benefited by knowing of the wide variation in numbers and percentages of eligible women in the state's counties. For instance, the county estimates inform decisions related to the dissemination of resources and funds to the community partnerships that assist the program with public education, outreach, and clinical quality assurance measures. The estimates by racial/ethnic group are useful in developing culturally appropriate messages and educational materials and in improving access to high quality screening services.

Other public health programs that have difficulty describing the distribution of their target populations because of a general lack of local data on health insurance status may also benefit from applying the method we have described. For example, other states that participate in the National Breast and Cervical Cancer Early Detection Program (NBCCEDP) (http://www.cdc.gov/cancer/nbccedp/) have eligibility criteria similar to those of California's CDP:EWC program and could produce meaningful estimates of eligible local populations by racial/ethnic and age groups by applying the small-area estimation method using a state survey or the Current Population Survey (a national survey that contains health insurance information [www.census.gov/cps/]) and census data (29). WISEWOMAN (Well-Integrated Screening and Evaluation for Women Across the Nation [www.cdc.gov/wisewoman/]), a state-based program offering NBCCEDP-enrolled women free or low-cost risk-factor screening, lifestyle interventions, and referral services aimed at preventing cardiovascular and other chronic diseases (30), could use this method to determine local estimates of the eligible population by demographic group to help identify provider sites and to determine the number of potential WISEWOMAN recruits.

One might think that in this age of information, data to describe any population of interest would be easy to obtain. This is not always the case, however, particularly when a population is narrowly defined, either by residence in a small geographic area or by specific characteristics. Small-area estimation statistics, as applied in our example, give public health programs a means of obtaining reliable estimates of their local or sparse target populations, even when no data seem to be available.

## Figures and Tables

**Table 1:** Covariates Included in the Final Model to Estimate the Proportion of Women Aged 40 Years or Older Eligible for Breast Cancer Screening and Diagnostic Services Through Cancer Detection Programs: Every Woman Counts, by County, California, 1998–2003

Covariates	Coefficient	Standard Error	*P* value
**Intercept**	-0.773	0.342	.03
**Race/ethnicity**			
Hispanic	Ref	Ref	Ref
White	-1.743	0.433	<.001
Black	-1.284	0.118	<.001
Asian/Pacific Islander	-0.588	0.418	.16
American Indian/Alaska Native	-0.027	0.661	.97
**Median household income**	-0.127	0.106	.23
**Unemployment rate**	-8.160	3.859	.03
**% Women living in poverty**	6.191	3.262	.06
**Interaction**			
Median household income X white	-0.271	0.139	.05
Unemployment rate X white	8.833	4.971	.08
Unemployment rate X Asian/Pacific Islander	-13.870	8.029	.08
% Women living in poverty X white	-5.816	4.231	.17
% Women living in poverty X American Indian/Alaska Native	-6.947	5.148	.17

Ref indicates reference group.

**Table 2:** Estimated Number and Proportion of Women Aged 40 Years or Older Eligible for Breast Cancer Screening and Diagnostic Services Through Cancer Detection Programs: Every Woman Counts, by County, California, 1998–2003

County	Service-Eligible Women	

	No.	% (95% CI)
Alameda	42,756	12.5 (12.1-12.9)
Alpine	39	11.8 (10.8-12.8)
Amador	1,021	9.6 (8.1-11.1)
Butte	7,539	13.6 (13.2-14.0)
Calaveras	1,490	10.6 (10.4-10.8)
Colusa	771	18.2 (17.7-18.6)
Contra Costa	23,184	9.1 (8.7-9.6)
Del Norte	1,034	15.6 (14.8-16.4)
El Dorado	3,773	8.0 (7.3-8.8)
Fresno	34,886	20.7 (20.4-21.0)
Glenn	1,065	17.3 (16.7-17.9)
Humboldt	4,400	13.9 (13.2-14.6)
Imperial	11,029	35.3 (35.0-35.6)
Inyo	760	14.1 (12.9-15.3)
Kern	29,354	19.7 (19.5-20.0)
Kings	4,223	18.1 (17.5-18.6)
Lake	2,932	15.9 (15.7-16.1)
Lassen	810	12.5 (12.1-13.0)
Los Angeles	436,334	20.0 (19.7-20.3)
Madera	5,163	17.3 (16.9-17.8)
Marin	4,101	5.5 (4.9-6.2)
Mariposa	641	12.1 (11.9-12.4)
Mendocino	3,224	13.9 (12.8-14.9)
Merced	8,354	18.6 (18.3-19.0)
Modoc	430	15.9 (15.4-16.3)
Mono	326	10.6 (10.0-11.2)
Monterey	12,580	14.8 (14.3-15.3)
Napa	3,704	10.8 (10.0-11.6)
Nevada	2,525	8.8 (8.2-9.3)
Orange	77,136	11.5 (11.1-11.8)
Placer	5,809	7.3 (6.6-7.9)
Plumas	711	11.2 (10.9-11.5)
Riverside	66,256	16.3 (16.1-16.6)
Sacramento	40,791	13.5 (13.0-13.9)
San Benito	1,555	13.2 (12.6-13.9)
San Bernardino	75,547	20.3 (20.0-20.6)
San Diego	94,834	14.4 (14.0-14.8)
San Francisco	24,506	13.0 (12.5-13.5)
San Joaquin	20,079	15.2 (14.9-15.6)
San Luis Obispo	7,451	11.0 (10.1-11.8)
San Mateo	16,469	9.1 (8.4-9.8)
Santa Barbara	12,788	13.9 (13.5-14.4)
Santa Clara	35,622	9.1 (8.5-9.7)
Santa Cruz	6,469	10.5 (10.3-10.7)
Shasta	6,024	12.8 (12.4-13.3)
Sierra	122	11.2 (10.5-12.0)
Siskiyou	2,007	14.7 (14.3-15.1)
Solano	10,432	10.9 (10.0-11.7)
Sonoma	10,952	8.7 (8.2-9.2)
Stanislaus	14,081	13.5 (13.0-14.0)
Sutter	2,411	12.3 (11.5-13.2)
Tehama	2,216	14.6 (14.3-14.9)
Trinity	595	14.9 (14.4-15.4)
Tulare	15,932	21.3 (21.0-21.5)
Tuolumne	1,806	11.0 (10.7-11.3)
Ventura	26,985	13.9 (13.5-14.4)
Yolo	5,731	15.1 (14.5-15.7)
Yuba	2,179	16.0 (15.8-16.2)
